# American Dental Association

**Published:** 1892-09

**Authors:** 


### AMERICAN DENTAL ASSOCIATION.

*August 2, 1892.—First Day.—Morning Session.*

MEETING called to order at 11 o'clock, with the President, Dr. W. W. Walker, in the chair.

The meeting was opened with prayer by Rev. Mr. Baker, of Niagara Falls.

The roll of membership was then called by the secretary, and a motion made to dispense with the reading of the minutes, which was carried.

### REPORTS OF OFFICERS.

*Dr. Crouse.*—The only report the Executive Committee has to offer is the programme, which has been distributed. It will be seen that we have left the afternoons open for clinics, extra meetings, etc. Other than that, it is not different from any other programme we have had. I will say also that the Executive Committee has been working vigorously all the year to get this audience here; we have sent out two sets of circulars to all the eligible members of every local society in the United States.

Report accepted.

REPORT OF PUBLICATION COMMITTEE.

*Dr. Cushing.*—The committee had made arrangements last year with the S. S. White Company to publish the transactions, and they were mailed January 1, 1892, and distributed to such societies, libraries, journals, and honorary members as were entitled to them. There are now left about seventy copies. I have also to report the following:

RECEIPTS AND DISBURSEMENTS.

| | | |
|---|---|---|
| Received from the treasurer | $120.50 | |
| Sale of transactions | 3.00 | |
| | | $123.50 |
| Expended for expressage, postage, etc. | $15.65 | |
| Postage | 8.00 | |
| For printing | 4.50 | |
| Engrossing memorial | 16.00 | |
| S. S. White Dental Company | 70.50 | |
| | | 114.65 |
| Leaving a balance due to the Association of | | $8.85 |

Referred to the Auditing Committee.

REPORT OF THE TREASURER, BY DR. FULLER.

| | | |
|---|---|---|
| Balance on hand August 4, 1891 | $1193.42 | |
| From dues to date | 1025.00 | |
| From money received from the Michigan State Society | 25.00 | |
| | | $2243.42 |
| Expenditures | | 1033.35 |
| Leaving a balance of | | $1210.07 |

Of this balance $300 is the unexpended portion of the $500 for Section VI., and $25 is the money from the Michigan State Society.

Report referred to the Auditing Committee.

The President, Dr. Walker, then read his address, of which the following is an abstract:

Gentlemen,—While I have but one or two special subjects to which I feel called upon to specifically direct your attention, it is proper, I think, that I should congratulate you upon the progress and high rank American dentistry has attained through the combined efforts of the members of this Association. . . . But while this is true, it is also true, judging the future by the past, that there yet remains a wide field for successful experiment and practical application in which and by which science, as the chief handmaid of our profession, shall yield results equally, if not indeed surpassing, the achievements hitherto attained.

THE WORLD'S COLUMBIAN DENTAL CONGRESS.

We are all aware of the joint action of the Southern and American Dental Associations, which created an Executive Committee of fifteen, clothed with full power to take such action as in its judgment it should deem best to carry out the objects of its organization, such action to be final and binding. . . . We stand pledged to do whatever may be in our power to make the occasion contribute notably to the elevation of the profession, to stimulate the spirit of research, to strengthen fraternal courtesy, and promote cardinal co-operation throughout the world among all who desire the advancement of dental science and art. The aim would be too limited if it included less than this, and the enterprise will be a failure if it falls short of a realization of this ambition.

It is the first time in the history of American dentistry that we have had an opportunity of demonstrating to the whole world what has been accomplished in all departments by American dentists in the last half-century. . . .

All reputable dentists of the United States are cordially invited to participate in this congress, and there is reason to believe that the invitation will be accepted by large numbers of foreign dentists. We expect the presence of distinguished representative dentists from almost every civilized country, and nearly all of note in our own, resulting in the largest gathering of dental practitioners in the history of our profession.

To accomplish this vast work a considerable sum of money will be needed. The General Finance Committee, which consists of Drs. L. D. Shepard, A. L. Northrop, and Truman W. Brophy, after careful estimates, place the amount to be contributed in advance of the congress at thirty thousand dollars; so you may imagine the magnitude of the work to be done by the twenty-three committees under the Executive Committee. . . .

DENTAL EDUCATION, LEGISLATION, AND THE DENTAL DEGREE.

With so many different colleges in the United States, most of them recognized as reputable, and about the same number of State examining boards, or boards of censors, as termed in some States, all working presumably for the welfare, advancement, elevation, and harmony of the dental profession, I feel justified, after a recent personal experience, in bringing these matters before this

Association for consideration, and, if deemed expedient, for some kind of action.

As the law stands to-day, students can enter any of the dental colleges with a certificate from a grammar-school, or after passing a preliminary examination in branches prescribed by the National Association of Dental Faculties, and pursuing a curriculum which will extend over three years, with an annual term of seven to nine months, and then passing the prescribed final examinations. Now, with all these qualifications which have been made necessary, and a degree granted by any of the reputable colleges, it would seem reasonable that a graduate from any such school should be allowed to practise his profession in any State of the Union without being put through the humiliation of another examination.

If the growing tendency towards dissociating the teacher's from the examining and licensing function should become the rule whereby college faculties shall be concerned solely with the educational training of students, and the work of examining and licensing be relegated to properly constituted and trustworthy State boards of examiners, there could be no valid excuse for the lack of inter-State comity in the matter of mutually recognizing State standards of dental education.

We should have more confidence in our fellow-men, and not degrade ourselves with suspicions of any lack of integrity on the part of the personal staff or faculty of a properly conducted and reputable college.

We must remember that the gentlemen composing such faculties are among the most honored members of our profession, and have worked with diligence and zeal to assist in making it what it is to-day. I will grant you that up to within a few years the faculties of our dental schools might in some instances have been a trifle more lenient with students than they should have been, and many were sent out into the world perhaps not quite up to the standard; but with the new dental law which has gone into effect we will have a new order of things, and active legislation tending to harmonization of the standards of the various State examining boards, so that the certificate of each will secure to the holder of it unrestricted license to practise in all the other States, is a question which immediately concerns us at this time, and is respectfully submitted for your action.

There has been too much clashing and slashing going on between schools and different State boards.

Now, gentlemen, this sort of thing should be stopped, and I desire to recommend a plan for the remedy. In an annual address before the Dental Society of the State of New York, delivered in May, 1891, I made the following suggestion: I have learned from reliable authority that several of our prominent colleges are willing that their candidates for graduation shall be examined by their respective State boards at the same time that the Faculty examinations take place, and when the candidate has passed both bodies satisfactorily, that the usual diploma shall be conferred, countersigned by the proper officers of the State examining board. I will say that these gentlemen are of the same opinion to-day.

In all States where dental colleges exist this plan should be adopted. I think all States should be divided into districts, each, possibly, with its corresponding district society, and each district should have a board of examiners, and in the State board each district should be represented by one examiner from the district organization, recommended to the State society by the said district, and all State boards so constituted by this system should be elected by the dentists in the State, and never appointed by the governor of the State.

A national board of examiners is probably not necessary, but if the need should arise such a board could easily be created under the scheme here proposed, and when so created should act only as a final court of appeal, with jurisdiction over questions involving lack of harmony in inter-State relations concerning matters of examinations, licenses, etc., and formulate plans tending to uniformity in matters of ethics, legislation, and education throughout the United States.

COMMITTEE ON STATE AND LOCAL ORGANIZATIONS.

The question of some practical method by which the members of the dental profession throughout the United States can be more definitely secured, and a closer relationship established between the American Dental Association and the various State and local organizations of this country, is one deserving of especial consideration by this body at this time. The best interests of American dentistry demand a more intimate and closely-knit organization of its best working forces, to the end that better work in all departments may be done, and especially is this necessary with reference to the showing we are to make at the coming World's Columbian Dental Congress. . . . It is proposed to emphasize the section work of the American Dental Association by making each section a repository of the results accomplished throughout the Union, in its given department, and that the annual report of the Association shall truly represent the progress of dentistry during the previous year. As a foundation for the accomplishment of this purpose it is suggested that a series of questions, carefully chosen as to importance and suggestiveness, though few in number, be formulated by this body, and circular-letter copies be sent to every State and local organization in America, with the request that one or more of the queries shall be taken up as a regular order of business for careful consideration and discussion at each stated meeting of such local organization to whom the circular is sent; that a careful and accurate report of the discussion on each topic shall be sent to the secretary of the American Dental Association, who shall transmit the report to the chairman of the proper section, who shall then, from all reports, so secured, collate the date contained therein and make a careful digest of the same, which shall constitute the basis of his report to the American Dental Association, for discussion by the whole body and publication in its annual report of transactions. As a part of this plan, each local organization, furnishing a report of its discussions, shall be requested to send its quota of delegates to represent the said local organization and intelligently discuss the questions at issue, as embodied in the report of his society, when it comes up for action in the American Dental Association. . . .

The plan here proposed is not intended to do away with that already in operation, . . . but it is suggested as an additional plan of work, by which it is hoped to secure several valuable factors, tending to increase the usefulness and strength of our Association. These are, briefly, 1, a more intimate relation and community of interest between the American Dental Association on the one hand, and the State and local organization on the other, and, growing out of this, 2, the co-operative organization of the whole working force of the dental profession in America; 3, the plan proposed will furnish the necessary material as a basis for the active development of the sectional work in this Association and place the sections on a firm basis, so that each will become a sub-organization engaged in special work under the government of the general body, which then will become a truly representative federation of active societies of specialists, and cease to be little more than an expansion of the usual local organization. . . .

For carrying out the purposes of the proposed plan, I would suggest that a standing committee be appointed, which shall be known as the Committee on State and Local Organizations, whose duty it shall be to secure the co-operation of all State and local organizations throughout the land . . . and have full charge of all matters which may properly fall within the limit of subjects indicated by its title. . . .

NEXT ANNUAL MEETING.

. . . If, as is expected, all reputable dentists in the United States will attend the World's Columbian Dental Congress, it will certainly be inexpedient, if indeed it will be possible, to hold two meetings so near the same time.

In the first place, the sections could not get material, and, secondly, we could not secure a full attendance. I would suggest that the next meeting be simply a business one, and that the only business to be transacted shall be the election of officers for 1894, and action upon the report of the special committee to whom was referred the amendment of the Consitution and By-Laws. . . .

Reference was then made to the death of Dr. John Allen, who died at his home in Plainfield, N. J., March 8, 1892.

*Dr. Patterson.*—I would suggest that the President's address be referred to a committee, and after the committee reports the discussion can take place.

On motion, the following committee was appointed: Drs. E. C. Kirk, L. D. Shepard, and J. N. Crouse.

*Dr. Crouse.*—At the end of this programme you will see printed certain rules which govern the speaking. One is in regard to the giving of the name before speaking, and the others about paying the dues. I hope all the members will follow those rules.

*Dr. Hunt.*—I would like to make an announcement: At the last meeting of the Executive Committee of the World's Columbian Dental Congress, some new appointments were made, and I would be pleased to meet the chairmen of the various sub-committees, to give them the additional names that have been placed on the committees, in order that all may be notified to attend the meetings.

*Dr. Crouse.*—As is probably known, the Dental Protective Association is taking testimony at the Cataract House during this week. If there are any members here who have testimony which they consider important, I will be very glad to receive it. It will save an immense amount of time and money, because I have to travel all over the country to get it. We have a test case on now, and we want to get all the testimony we can. I also have some by-laws in my pocket which are not signed, and if any one wishes to join the Association I shall be glad to take his name.

On motion, the secretary was authorized to pay all properly authenticated bills.

*Dr. Peirce.*—The Committee on Voluntary Essays wishes to report that it has received one paper from Dr. Stainton, of Buffalo, entitled "Crownless Teeth."

